# Polymorphism rs13334190 in zinc finger protein 469 (*ZNF469*) is not a risk factor for keratoconus in a Saudi cohort

**DOI:** 10.1186/s13104-017-2996-8

**Published:** 2017-11-29

**Authors:** Hatem Kalantan, Altaf A. Kondkar, Tahira Sultan, Taif A. Azad, Nasser A. Alsabaani, Masoud Ali AlQahtani, Abdulrahman Almummar, Yuato Liu, Khaled K. Abu-Amero

**Affiliations:** 1 Glaucoma Research Chair, Department of Ophthalmology, College of Medicine, King Saud University, Riyadh, 11411 Saudi Arabia; 2 Ophthalmology Department, College of Medicine, King Khalid University, P.O. Box 641, Abha, 61421 Saudi Arabia; 3 Assir Central Hospital, P. O. Box 35, Abha, 61421 Saudi Arabia; 4 Augusta University, 1460 Laney Walker Blvd, CB1123, Augusta, GA 30912 USA; 5 Department of Ophthalmology and Visual Sciences, University of Illinois at Chicago, Chicago, IL 60612 USA; 6 Ophthalmic Genetics Laboratory, Department of Ophthalmology, College of Medicine, King Saud University, P.O. Box 245, Riyadh, 11411 Saudi Arabia

**Keywords:** Saudi, Keratoconus, rs13334190, *ZNF469*

## Abstract

**Objective:**

Polymorphism rs13334190 in the zinc finger protein 469 gene has been suggested to predispose toward a “thin” cornea, which then becomes keratoconic or is directly pathogenic. Thus, we genotyped polymorphism rs13334190 in 127 unrelated keratoconus cases and 168 control subjects from Saudi Arabia using Taq-Man^®^ assay.

**Results:**

The genotype frequency distribution did not deviate significantly from the Hardy–Weinberg equilibrium (*p* > 0.05). Overall, both the genotype and allele frequencies were not significantly different between cases and controls. A minor allele frequency of 0.068 was comparable to the aggregate rates ranging from 0.060 to 0.086 observed in other populations. Binary logistic regression analysis was performed to ascertain the effects of age, gender and genotype on the likelihood of having keratoconus. The analysis indicated that increased age was statistically significant (p = 0.000) and that females have a 2.19-fold increased risk (p = 0.018) of developing keratoconus. The genotype frequencies did not differ between the sporadic or familial keratoconus cases. Polymorphism rs13334190 is not an independent risk factor for keratoconus in the Saudi cohort.

## Introduction

Keratoconus (KTCN; OMIM 148300) is a non-inflammatory corneal ectatic disease resulting in steepening and distortion of the cornea, altered refractive powers and altered visual acuity. Advanced cases of KTCN exhibit corneal scarring due to corneal edema and decompensation that may further reduce the visual acuity. The clinical symptoms of KTCN are highly variable and depend upon the stage of progression of the disease [1]. The prevalence of KTCN has been reported to vary among different studies, ranging from 0.3 per 100,000 in Russia to 2300 per 100,000 in central India [2] and this variation is in part due to the differences in the applied diagnostic criteria and geographic location. The disease occurs with no ethnic or gender preponderance and causes significant visual impairment [2].

Increased evidence suggests an involvement of a genetic component in disease susceptibility. This hypothesis is getting widely accepted, especially with the predominance of KTCN among certain families, twins and particular ethnicities [3]. Accordingly, family history is an important risk factor and obtaining a thorough family medical history is crucial when considering a refractive surgery. Various candidate single nucleotide polymorphisms (SNPs), genes and copy number variations have been associated with the susceptibility to KTCN [4]. In addition, mitochondrial abnormalities have also been reported [5]. Reactive oxygen species are a known contributor to tissue damage and can be a significant player in the characteristic corneal thinning and ectasia that lead to the development and progression of KTCN [6]. KTCN cases exhibiting both autosomal recessive and dominant patterns of inheritance have been documented [3]. There are several chromosomal loci and genes reported in association with KTCN [7, 8], some of which were eventually excluded [7, 9], while others showed no confirmed association with the disease [10, 11]. The major genetic contributor to KTCN, however, is yet to be discovered.

In 2014, Vincent et al. [12] screened 43 Polynesian KTCN patients for mutations in the zinc finger protein 469 (*ZNF469*) gene. They detected three heterozygous variants, which were predicted to be pathogenic and suggested that these variants are either genetically predisposed toward a “thin” cornea, which then becomes keratoconic or are directly pathogenic. One of those variants is the p.R2129K, which is the subject of our investigation [12]. Lechner and colleagues [13] found a significant enrichment of potentially pathologic heterozygous alleles in *ZNF469* associated with the development of KTCN (*p* = 0.00102) resulting in a relative risk of 12.0. This enrichment of the potentially pathogenic alleles in *ZNF469* in 12.5% of KTCN patients represents significant mutational load and highlights *ZNF469* as the most significant genetic factor responsible for KTCN identified to date. In contrast, Karolak et al. [14] found that the high prevalence of *ZNF469* variants identified in Polish KTCN patient is typical for a common genetic variation observed in the general population. Therefore, authors concluded that variations in the *ZNF469* gene are not responsible for KTCN in their population. Here, we tested whether SNP rs13334190 (p.R2129K) variation in the *ZNF469* gene is associated with KTCN in patients from Saudi Arabia.

## Main text

### Methods

The study adhered to the tenets of the Declaration of Helsinki and was approved by the College of Medicine (King Saud University, Riyadh, Saudi Arabia) institutional review board committee (proposal number #09–659). Written informed consent was obtained from all participants. KTCN patients (n = 127) were selected from the anterior segment clinic at King Abdulaziz University Hospital, Riyadh, Saudi Arabia. Diagnosis of KTCN was confirmed if the Schimpff-flow based elevation map showed posterior corneal elevation with the central 5 mm ≥ + 20 µm, inferior–superior dioptric asymmetry (I–S value) > 1.2 diopters (D) and the steepest keratometry > 47 D. Identification of an isolated sporadic case or familial KTCN was done after examining the immediate family members. Individuals showing presence of post-laser-assisted in situ keratomileusis ectasia and secondary causes of KTCN such as trauma, surgery, Ehlers Danlos syndrome, osteogenesis imperfecta and pellucid marginal degeneration were excluded. Control subjects (n = 168) had no ocular disease(s) or previous ophthalmic surgeries on clinical examination. They had normal slit lamp cornea and Schimpff-flow based elevation map. Subjects refusing to participate in this study were excluded.

#### DNA preparation

DNA was extracted from EDTA blood samples using the illustra blood genomicPrep Mini Spin kit (Catalog No. 28904265, GE Healthcare, Buckinghamshire, UK) and stored at – 20 °C in aliquots until further use.

#### Genotyping of rs13334190 in *ZNF469*

DNA samples were genotyped for polymorphism rs13334190 using the TaqMan^®^ SNP genotyping assay (Applied Biosystems Inc., Foster City, CA, USA) on ABI 7500 real-time PCR system (applied biosystems) as described previously [15]. For genotyping rs13334190 polymorphism, assay ID: C_30944445_10 (applied biosystems) was used. A 25 µL PCR reaction consisted of 1X TaqMan^®^ genotyping master mix (applied biosystems), 1X SNP genotyping assay mix and 20 ng DNA. Each 96-well plate included two no DNA negative controls. The recommended PCR conditions included incubation at 95 °C for 10 min, followed by 40 cycles, denaturation at 92 °C for 15 s and annealing/extension at 60 °C for 1 min. The VIC^®^ and 6-carboxy-fluorescein (FAM) fluorescence levels of the PCR products were measured at 60 °C for 1 min. Genotype calling was performed using the automated 2-color allele discrimination software on ABI 7500.

#### Statistical analysis

Independent sample’s *t* test (2-tailed) was used to test difference between cases and controls in terms of continuous variables. Pearson’s Chi^2^ test was used to test Hardy–Weinberg equilibrium (HWE) deviation and detect any association between the different genetic profiles. Risk was expressed as odds ratio (OR) with 95% confidence interval (CI) level. A *p* value less than 0.05 were considered statistically significant. All the analysis was done using SPSS version 22 (IBM Inc. Chicago, Illinois, USA).

### Results

The demographic and other characteristics of subjects included in this study are shown in Table 1. A total number of 295 subjects, including, 127 cases of KTCN and 168 controls were genotyped in this study. KTCN cases included both isolated, sporadic cases (n = 85) and familial cases (n = 42). The results presented in Table 1, indicate that the patient group was significantly younger than the control group with age ranging from 12 to 50 year and 20 to 90 year, respectively. In addition, the gender distribution between the two study group was also found to be significantly different (*p* = 0.010).

**Table 1:** Demographic characteristics in keratoconus patients and controls

Demographic characteristics	Patients (n = 127) No. (%)	Controls (n = 168) No. (%)	*p* value (2-tailed)
Age in years, mean (± SD)	26.9 (6.6)	46.9 (15.2)	0.000*
Male	65 (51.1)	111 (66.0)	0.010^§^
Female	62 (48.8)	57 (33.9)	–
Familial keratoconus	42 (33)	–	–

* *t* test, ^§^ Pearson Chi^2^ test

The distribution of rs13334190 polymorphism did not deviate significantly from the HWE (*p* > 0.05). Association analysis between cases and controls revealed no significant genotype distribution under additive (Chi^2^ = 1.90, df = 2, *p* = 0.387) and dominant models (Chi^2^ = 1.43, df = 1, *p* = 0.232). Besides, the allele frequency distribution was also non-significant (OR = 0.616; 95% CI = 0.29–1.28; *p* = 0.194). The minor “A” allele frequency was 0.043 and 0.068 among KTCN patients and controls, respectively (Table 2). In addition, to ascertain the effects of age, gender and genotype on the likelihood of having KTCN a binary logistic regression analysis was performed. The analysis indicated that increased age was statistically significant (p = 0.000) and that females were having a 2.19-fold increased risk (p = 0.018) of developing KTCN (Table 3). Furthermore, the distributions between sporadic (n = 85) and familial cases (n = 42) were similar. The homozygous genotype was absent, whereas, the heterozygous genotype G/A was 9.5 and 9.4% in familial and sporadic KTCN cases, respectively.

**Table 2:** Association analysis of rs13334190 in *ZNF469* gene with keratoconus

SNP (gene)	rs13334190 (*ZNF469*)				
Allelic analysis	Patients (n = 127) No. (%)	Controls (n = 168) No. (%)	Odds ratio	95% confidence interval	*p* value^a^ (2-tailed)
G	243 (0.956)	313 (0.931)	1	Reference	–
A^b^	11 (0.043)	23 (0.068)	0.616	0.294–1.288	0.194
HWE p	0.61	0.79	–	–	–
Genotype and model analysis					
G/G	116 (91.3)	146 (86.9)	1	Reference	–
G/A	11 (8.6)	21 (12.5)	0.659	0.30–1.42	0.285
A/A	0 (0)	1 (0.6)	–	–	–
Additive (trend)	–	–	–	–	0.387
Dominant	–	–	0.629	0.29–1.35	0.232

^a^Pearson Chi^2^ test

^b^Risk variant, *HWE p* Hardy–Weinberg equilibrium *p* value

**Table 3:** Effect of age, sex and genotype on disease outcome by binary logistic regression analysis

Variables	Odds ratio	95% confidence interval	*p* value (2-tailed)
Age	0.83	0.80–0.87	0.000
Sex^a^	2.19	1.14–4.18	0.018
Dominant model^b^	0.76	0.27–2.16	0.612

^a^Male as reference

^b^G/G as reference

### Discussion

The SNP rs13334190 in the *ZNF469* was previously reported in association with thin cornea and KTCN. Here, we screened a group of KTCN patients and controls from the Saudi Arab ethnicity. The heterozygous and homozygous mutant genotype distribution was similar and did not yield significant statistical difference. Similarly, the minor allele frequency was similar in the two study groups, indicating that the allele and mutant genotypes are not an independent risk factor for KTCN in this population. This finding is similar to previous studies, which could not establish a link between rs13334190 and KTCN in the Polish population [14]. In the same report, Karolak and colleagues [14] reported that this SNP is a benign polymorphism diminishing its pathogenic significance. In contrast, the rs13334190 SNP was predicted to be pathogenic in a cohort of New Zealand despite the lack of clear explanation of its role in KTCN-pathogenicity and its Polynesian controls at a rate > 1% [12]. Lechner and colleagues [13] found a significant association between this SNP and KTCN in a European cohort from the UK and Switzerland.

In our KTCN cohort of Saudi origin (n = 127), 42 (33.1%) were familial cases, whereas 85 (66.9%) were sporadic. The prevalence of high percentage of familial KTCN cases is unusual and may be reflected by the high consanguineous marriages in Saudi Arabia as we reported previously [16]. When we investigated the genetic difference for SNP rs13334190 in familial and sporadic cases, we did not find any difference. Higher familial cases in our population may indicate an autosomal recessive gene(s) yet to be detected.

The minor ‘A’ allele frequency found in Saudi controls is 0.068, which is comparable to rates observed in various populations, which ranged from 0.060 to 0.086 (http://www.ensembl.org). In contrast, the minor allele frequency in Polynesian population was 0.14 and this clearly reflects an ethnicity-based variation. A higher rate of the minor allele in the Polynesians as compared to others may be reflected by the fact that Polynesian is an Islander population and may be regarded as genetically isolated population.

The study provides no evidence of any association between SNP rs13334190 in *ZNF469* gene and KTCN patients of Saudi origin.

### Limitations

The number of patients with KTCN and controls in this study was relatively small as was the sample size in each subgroup. Therefore, the lack of significance in our study could be due to relatively small sample size and insufficient statistical power of this study cohort. Population-based replication studies in different ethnic groups are necessary to identify and/or confirm genetic contribution of rs13334190 or other SNP(s) in *ZNF469* to KTCN.

## Abbreviations

CI: confidence interval
HWE: Hardy–Weinberg equilibrium
KTCN: keratoconus
OR: odds ratio
SNP: single nucleotide polymorphism
*ZNF469*: zinc finger protein 469
